# Comparative architectural evaluation of CNN models with alternative classifiers for UAV-based detection of damaged green zones

**DOI:** 10.3389/frai.2026.1910842

**Published:** 2026-10-28

**Authors:** Nodir Rakhimov, Jura Kuvandikov, Ma'ruf Tojiyev, Dilmurod Khasanov, Ravshan Shirinboyev, Kholmurot Rakhimov, Khakim Alimov, Rashid Nasimov

**Affiliations:** 1 Software of Information Technologies, Tashkent University of Information Technologies Named After Muhammad Al-Khwarizmi, Tashkent, Uzbekistan; 2 Department of Computer Science and Programming, Jizzakh Branch of the National University of Uzbekistan Named After Mirzo Ulugbek, Jizzakh, Uzbekistan; 3 Department of Management Theory and Information Security, Samarkand State University Named After Sharof Rashidov, Samarkand, Uzbekistan; 4 Department of Mathematics, University of Economics and Pedagogy, Qarshi, Uzbekistan; 5 Department of Mathematics, Jizzakh State Pedagogical University, Jizzakh, Uzbekistan; 6 Department of Artificial Intelligence, Tashkent State University of Economics, Tashkent, Uzbekistan; 7 Department of Radioelectronic devices and systems, Tashkent State Transport University, Tashkent, Uzbekistan

**Keywords:** architectural evaluation, convolutional neural network, damaged vegetation detection, feature extraction, green zone monitoring, hybrid classification, UAV imagery, XGBoost

## Abstract

Unmanned aerial vehicle imagery provides a practical and high-resolution data source for monitoring the spatial condition of urban and peri-urban green zones. However, the reliable detection of damaged, stressed, or declining vegetation areas remains challenging because UAV images often contain complex background patterns, illumination variations, shadows, mixed vegetation structures, and visually similar damage symptoms. Convolutional neural networks have shown strong potential for extracting hierarchical visual features from image data, yet their conventional fully connected classification heads may not always provide the most robust decision boundary, particularly when training data are limited, class distributions are imbalanced, or subtle vegetation damage patterns must be separated. This study presents a comparative architectural evaluation of CNN-based models in which the internal components of the network, including convolutional kernel size, pooling strategy, flattening mechanism, and classification head, are systematically modified and assessed. A custom CNN architecture is used as the feature extractor, while its conventional classification head is replaced with alternative machine learning classifiers, including XGBoost, Random Forest, Support Vector Machine, and Logistic Regression. In addition, the effects of different convolutional kernels, max and average pooling operations, flatten and global average pooling representations are experimentally investigated. The intermediate outputs of convolution, pooling, and feature extraction stages are visualized to interpret how architectural modifications influence the representation of vegetation texture, color degradation, canopy gaps, and damaged regions. The proposed experimental framework enables a detailed comparison between conventional CNN classifiers and hybrid CNN–machine learning models. The study evaluates each configuration using classification metrics, visual feature analysis, and model interpretability tools. The outcome is to identify an effective CNN-based architecture for UAV imagery that improves damaged green zone detection while providing a clearer understanding of how architectural choices affect model behavior.

## 1 Introduction

Green zones play a crucial role in maintaining ecological balance, regulating the urban microclimate, mitigating urban heat island effects, supporting biodiversity, and improving the environmental quality and resilience of cities (Bowler et al., 2010; Herath and Bai, 2024). However, the condition of green areas is increasingly affected by water scarcity, soil degradation, pest pressure, air pollution, irregular irrigation, climate variability, and anthropogenic stress. In urban, peri-urban, and forest environments, vegetation decline may appear as localized damaged patches, canopy thinning, discoloration, dry or dead vegetation, and structural changes in the canopy. For example, UAV-based studies have been used to monitor forest health, identify standing dead trees, and detect disease-related changes in tree crowns (Ecke et al., 2022; Jiang et al., 2023; Makhmudov et al., 2026).

Unmanned aerial vehicle imagery has become a practical and effective tool for environmental monitoring because UAV platforms can acquire very-high-resolution images at flexible flight times and relatively low operational cost (Ecke et al., 2022). Recent studies on UAV remote sensing show that UAV-based data are increasingly used for classification, regression, vegetation monitoring, crop assessment, and plant-level analysis because they provide detailed spatial information that is often unavailable in conventional satellite imagery (Osco et al., 2021; Li et al., 2025; Shirinboyev et al., 2026). In green zone monitoring, UAV images make it possible to observe fine-scale variations in canopy structure, vegetation cover, color degradation, gaps, shadows, and localized damage patterns. These characteristics are particularly important when the objective is not only to classify vegetation and non-vegetation areas, but also to distinguish healthy, stressed, damaged, and declining vegetation conditions (Jiang et al., 2023). Despite these advantages, the automated detection of damaged green zones from UAV imagery remains a challenging computer vision problem.

UAV images often contain heterogeneous backgrounds, mixed vegetation species, variable illumination, cast shadows, complex canopy geometry, exposed soil, roads, rocks, and other non-vegetation elements, which can complicate vegetation-condition classification (Osco et al., 2021; Sandoval-Pillajo et al., 2025; Ecke et al., 2022). In addition, damaged vegetation may exhibit only subtle visual differences from healthy or moderately stressed vegetation, particularly in terms of color, canopy texture, and local structural changes, making reliable class separation challenging (Sandoval-Pillajo et al., 2025; Jiang et al., 2023; Makhmudov et al., 2026).

Convolutional neural networks have become one of the most effective deep learning approaches for image-based remote sensing analysis (Nogueira et al., 2017). CNN models have been widely investigated because they can capture spatial patterns, local textures, object boundaries, and high-level visual structures without relying entirely on manually engineered features (Kattenborn et al., 2021). In UAV imagery, CNN-based methods are especially valuable because very-high-resolution images contain detailed spatial information, and CNN filters can learn local visual cues such as leaf texture, canopy density, discoloration, bare soil exposure, and damaged vegetation patterns (Kattenborn et al., 2019; Makhmudov et al., 2026).

A conventional CNN architecture usually consists of convolutional layers, activation functions, pooling operations, flatten or global pooling layers, fully connected layers, and a final Softmax classifier (Nasimov and Cho, 2025). The convolutional part extracts visual features, while the fully connected part performs the final classification. However, the fully connected classification head may not always be the most effective decision module for UAV-based vegetation damage detection.

Previous image classification studies have shown that CNN–XGBoost models can be constructed by using CNN for automatic spatial feature extraction and XGBoost for feature classification (Jiao et al., 2021). Nevertheless, replacing the fully connected layer alone is not sufficient for a complete architectural understanding of CNN-based UAV image analysis. The performance of a CNN model is also influenced by convolutional kernel size, pooling strategy, feature dimensionality, flattening mechanism, and the depth of the feature extractor. Therefore, a systematic investigation of these architectural components is necessary to understand which CNN design choices are more appropriate for UAV-based damaged vegetation detection.

Existing studies on UAV vegetation monitoring and deep learning have mainly focused on selecting a single CNN architecture or improving classification accuracy using a specific model. Reviews of deep learning in UAV remote sensing and vegetation analysis indicate that CNN-based approaches are widely used for classification, regression, and vegetation mapping tasks (Osco et al., 2021). However, fewer studies provide a detailed comparative analysis of how internal CNN components and alternative classification heads affect the detection of damaged green zones. In particular, there is still a need for an experimental framework that jointly evaluates kernel configurations, pooling operations, flattening strategies, CNN feature extractors, and external classifiers under the same UAV imagery-based vegetation damage detection task. Figure 1 summarizes the overall research workflow.

**Figure 1:**
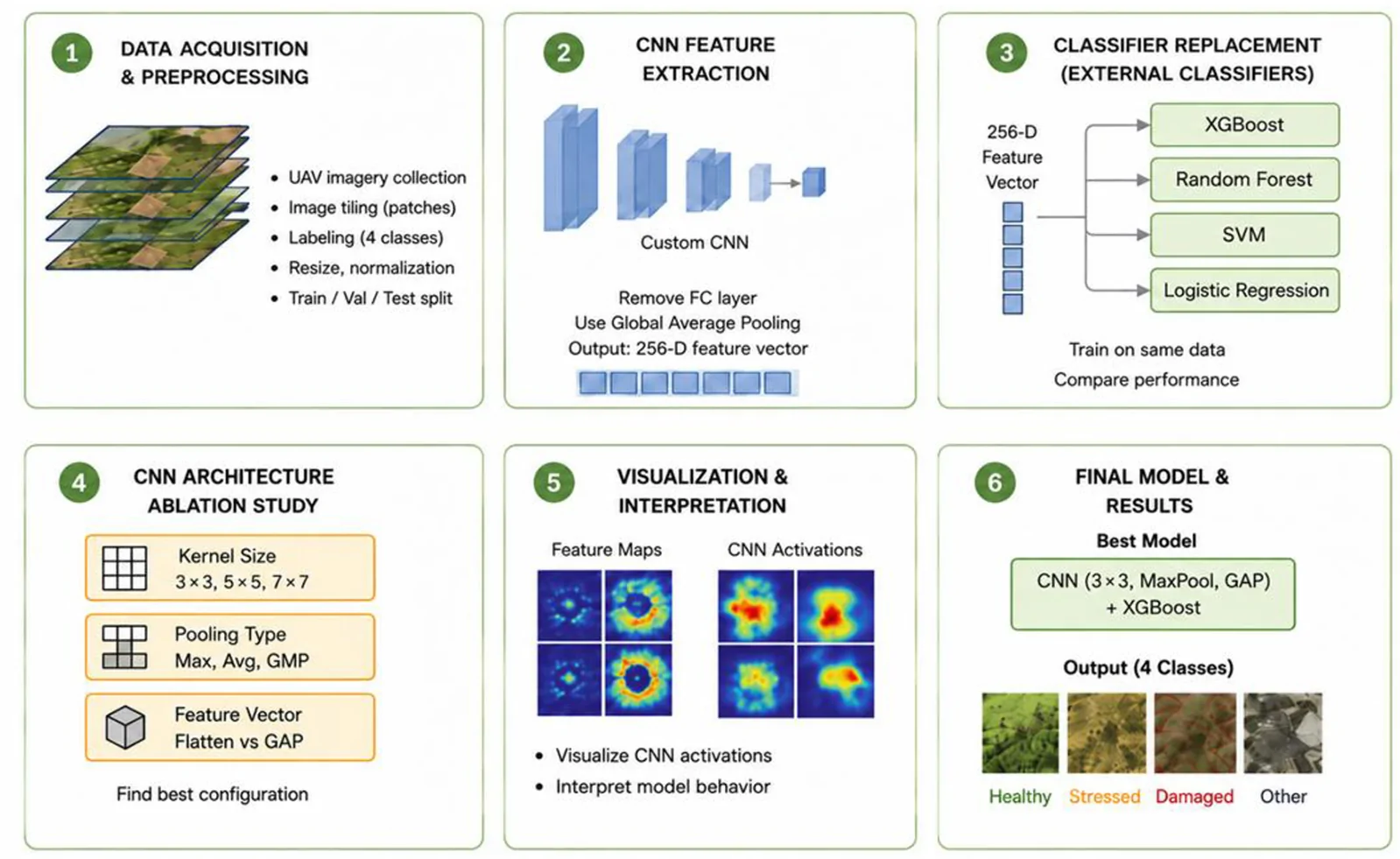
Basic concept and stages of research.

## 2 Related works

### 2.1 Deep learning in remote sensing image analysis

Zhang et al. (2016) reviewed early applications of deep learning in remote sensing image analysis and described how CNNs and other deep models were used for image classification and interpretation. Their study provided a methodological foundation for CNN-based feature extraction, but did not address replacing fully connected layers with external classifiers. Cheng and Han (2016) reviewed object detection methods in optical remote sensing images, including template matching, knowledge-based methods, object-based image analysis, machine learning, and early deep learning approaches. Although their study focused mainly on object detection, its discussion of feature representation is relevant to UAV-based green zone damage detection, where damaged vegetation patches may appear as small and irregular objects in high-resolution imagery.

A comprehensive survey of deep learning theories, tools, and challenges in remote sensing was presented by Ball et al. (2017), with particular attention to CNN-based models, limited labeled data, sensor heterogeneity, model interpretability, and training complexity. These issues are also relevant to UAV-based damaged green zone detection, where annotated samples are often limited and reliable interpretation of model decisions is required. Remote sensing image scene classification and the NWPU-RESISC45 benchmark dataset were investigated by Cheng et al. (2017), who emphasized the effects of scale, viewpoint, illumination, background, and spatial resolution variations on classification performance. Although their study focused on scene-level classification, these challenges are also relevant to UAV-based green zone analysis, where flight altitude, shadows, vegetation density, and mixed background objects can influence damaged vegetation detection. A comprehensive review and resource list on deep learning in remote sensing was presented by Zhu et al. (2017), covering classification, detection, segmentation, and other remote sensing tasks. Their discussion of deep models as potential black-box systems is relevant to the present study, since the proposed framework includes feature map, pooling output, and CNN representation visualization to better interpret damaged vegetation detection.

Deep learning approaches for both pixel-wise and scene-wise remote sensing image classification were reviewed by Li et al. (2018), who summarized common model types and publicly available datasets used in this field. Ma et al. (2019) conducted a meta-analysis of deep learning applications in remote sensing and showed the increasing use of CNN-based approaches across image classification, object detection, and land-cover analysis tasks. Their findings further support the effectiveness of deep feature learning for complex remote sensing imagery. Osco et al. (2021) reviewed deep learning applications in UAV remote sensing across environmental, urban, and agricultural tasks. However, their work did not experimentally examine CNN architectural components or classifier replacement strategies for vegetation damage detection. Figure 2 illustrates the conventional CNN feature-extraction and classification pipeline.

**Figure 2:**
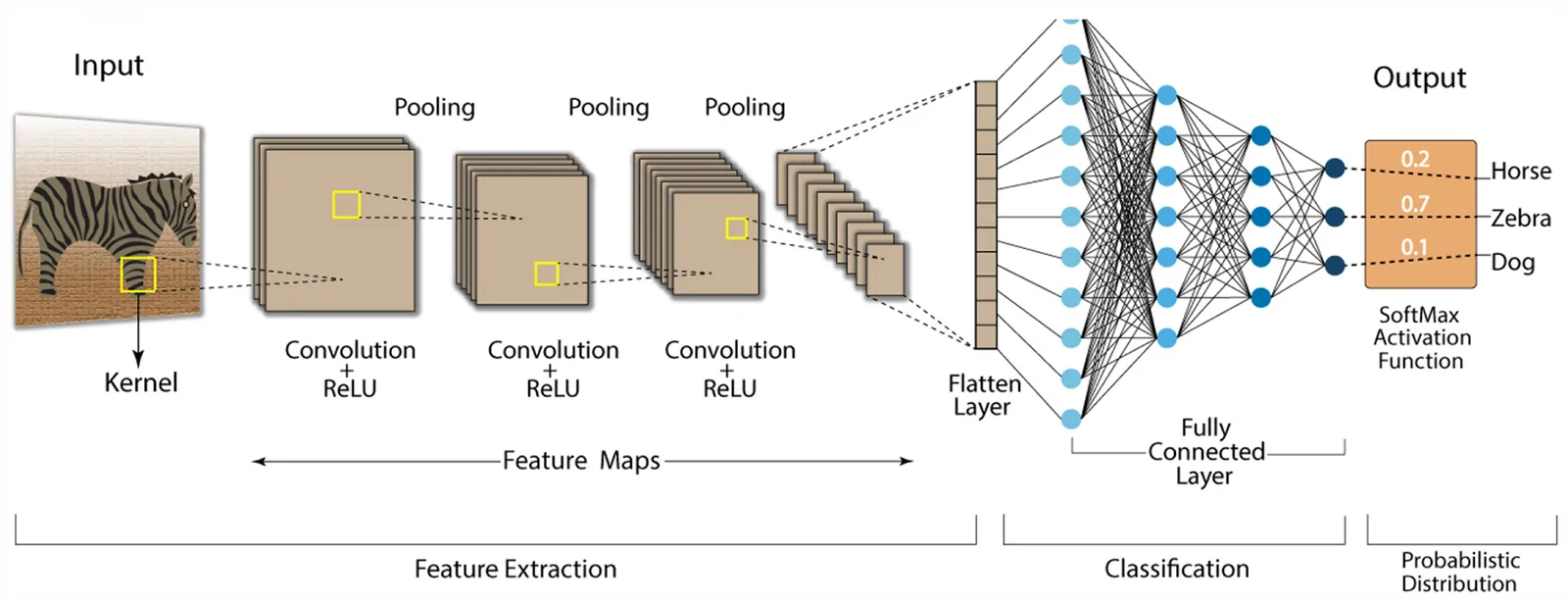
Typical convolution neural network architecture for classification (Nasimov and Cho, 2025).

### 2.2 Hyperspectral and UAV-based vegetation monitoring studies

Deep learning methods for hyperspectral image analysis were reviewed by Petersson et al. (2016), with emphasis on CNN and autoencoder-based feature extraction and classification (Nogueira et al., 2017). Hu et al. (2015) demonstrated the effectiveness of deep convolutional neural networks for hyperspectral image classification, showing that hierarchical CNN feature extraction can capture discriminative spectral-spatial representations. Although their study focused on hyperspectral imagery, it provides methodological support for the use of deep CNN-based feature extraction in remote sensing classification tasks. Adão et al. (2017) reviewed UAV-based hyperspectral sensors, data processing techniques, and applications in agriculture and forestry. Their study emphasized that UAV platforms equipped with hyperspectral sensors can provide detailed information about vegetation condition, plant stress, and forest or crop monitoring. This work is relevant because it supports the importance of UAV-based data acquisition for vegetation health assessment.

A multidisciplinary review by Signoroni et al. (2019) summarized deep learning applications in hyperspectral image classification, object detection, segmentation, denoising, super-resolution, and data fusion. Their work supports the value of deep feature extraction for complex remote sensing data, while the present study further compares CNN feature extractors with external classifiers for UAV-based green zone damage detection. Deep learning classifiers for hyperspectral imaging were reviewed by Paoletti et al. (2019), who discussed the strengths and limitations of different HSI classification methods. While their work highlights the difficulty of classifying complex high-dimensional remote sensing data, the present study focuses on UAV image patches, CNN architectural components, and replacing fully connected heads with machine learning classifiers. A comparative review of deep learning for hyperspectral data classification was presented by Audebert et al. (2019), emphasizing challenges such as spectral dimensionality, limited training samples, spatial resolution, and model transferability. Unlike their review, the present study experimentally evaluates CNN feature extractors, pooling strategies, flattening mechanisms, and external classifiers within a unified UAV-based damaged vegetation detection framework. Hybrid deep-learning approaches have also been applied to related forest-monitoring tasks, including early forest-fire detection (Mamadmurodov et al., 2025), illustrating the broader use of deep models for UAV- and image-based environmental monitoring.

### 2.3 CNN architectures and feature extraction models

ResNet was proposed by He et al. (2016) as a residual learning framework that enables very deep CNNs to be trained more effectively through skip connections. The Inception architecture introduced by Szegedy et al. (2015) uses multi-scale convolutional processing to capture image patterns at different spatial levels. This principle is relevant to UAV-based vegetation damage detection, where both local texture changes and broader canopy degradation may need to be represented. Sandler et al. (2018) proposed MobileNetV2, which uses inverted residual blocks and linear bottlenecks to build lightweight neural networks. Tan and Le (2019) introduced EfficientNet, which uses compound scaling to balance network depth, width, and input resolution, providing an efficient approach to CNN architecture design. This architecture is important for UAV-based monitoring systems because practical green zone assessment may require models that can run efficiently on limited hardware or near-real-time platforms (Tojiyev et al., 2025).

### 2.4 XGBoost and CNN–machine learning hybrid models

Chen and Guestrin (2016) introduced XGBoost as a scalable tree boosting system based on regularized gradient boosting. The method includes sparsity-aware learning, approximate tree construction, regularization, and efficient computation. XGBoost is relevant to the proposed study because CNN-extracted features can be converted into a structured feature vector and then classified using a regularized decision tree ensemble. This makes XGBoost a suitable replacement for the fully connected classification head of CNN models, particularly when non-linear feature interactions are important. Ren et al. (2017) proposed a hybrid CNN–XGBoost image-classification framework in which CNN was used for automatic feature extraction and XGBoost served as the final classifier. Their model was evaluated on the MNIST and CIFAR-10 datasets and achieved classification accuracies of 99.22% and 80.77%, respectively. For comparison, the corresponding conventional CNN results reported in the same study were 98.80% on MNIST and 76.28% on CIFAR-10, indicating that replacing the conventional CNN classifier with XGBoost improved performance on both benchmark datasets.

Jiao et al. (2021) further developed this approach by optimizing the CNN–XGBoost framework using adaptive particle swarm optimization (APSO). Their APSO-CNN-XGBoost model achieved accuracies of 99.60% on MNIST, 91.98% on CIFAR-10, and 66.35% on CIFAR-100. These results were higher than the corresponding PSO-CNN-XGBoost results of 99.41%, 91.21%, and 64.58%, respectively. Although these benchmark datasets differ substantially from UAV-based vegetation imagery, the reported results provide quantitative evidence that CNN feature extraction combined with XGBoost can improve classification performance and that optimization of the hybrid framework can further affect the final accuracy.

More recently, Kuang et al. (2026) proposed a CNN–LSTM–XGBoost hybrid framework for UAV-based nitrogen stress classification, further demonstrating the applicability of hybrid deep feature extraction and XGBoost-based classification in vegetation-related UAV imagery.

In addition to XGBoost, other machine learning classifiers are often used after deep feature extraction. Support Vector Machine can be effective when CNN features are separable in a transformed feature space. Random Forest can capture non-linear relationships using multiple decision trees and can provide feature importance. Logistic Regression can serve as a simple linear baseline. In the present study, these classifiers are not treated as independent alternatives to CNN, but as replacement heads that receive CNN-generated feature vectors.

### 2.5 Research gap

The reviewed studies indicate that deep learning has been widely applied in remote sensing, UAV imagery, hyperspectral image analysis, vegetation monitoring, scene classification, and object detection; however, several important gaps remain. Earlier surveys established the general role of deep learning in remote sensing, UAV-focused studies demonstrated the importance of UAV-based image acquisition for environmental and agricultural monitoring, CNN architecture studies provided strong feature (Huang et al., 2017) extraction backbones, and CNN–XGBoost works showed the potential of replacing neural classification heads with XGBoost. Nevertheless, most existing studies use CNNs as complete end-to-end models and do not sufficiently analyze the effects of internal architectural components such as kernel size, pooling type, flattening mechanism, and global average pooling. In addition, the fully connected classification head is often accepted as a default component, while its replacement with external classifiers has not been systematically compared for UAV-based vegetation damage detection. Therefore, the present study addresses these gaps by developing a comparative experimental framework that evaluates the effects of convolutional kernel size, pooling strategy, feature aggregation, and alternative classification heads within a custom CNN framework, modifies convolutional and pooling structures, compares flatten and global average pooling representations, replaces fully connected layers with external classifiers, and visualizes intermediate image transformations to explain why particular CNN–classifier configurations are more suitable for damaged green zone analysis. Table 1 summarizes representative related studies and their relevance to the present work.

**Table 1:** Summary of representative related works on deep learning, UAV imagery, CNN architectures, and hybrid CNN–classifier models.

References	Research focus	Methodological approach
Zhang et al. (2016)	Deep learning for remote sensing	CNN, DNN, autoencoder-based analysis
Ball et al. (2017)	Deep learning challenges in remote sensing	Survey of CNN-based remote sensing
Osco et al. (2021)	UAV remote sensing applications	DL-based classification and regression
Adão et al. (2017)	UAV hyperspectral vegetation monitoring	Hyperspectral sensing and data processing
He et al. (2016)	Deep CNN feature extraction	ResNet with residual skip connections
Ren et al. (2017)	Hybrid CNN–XGBoost image classification	CNN features with XGBoost classifier

## 3 Materials and methods

### 3.1 Dataset and UAV image acquisition

The dataset used in this study comprises 11,200 RGB UAV image samples obtained from the database of the Research Institute of Rainfed Agriculture of Republic of Uzbekistan. The authors accessed an existing institutional dataset rather than conducting a new UAV acquisition campaign for this study. The imagery represents forest farm areas and sloping mountain areas, including dense vegetation, irregular terrain, exposed soil, rocks, shadows, and mixed background objects. The dataset contained 11,200 original, non-augmented RGB image patches derived from the acquired high-resolution UAV photographs. Data augmentation was applied only after the dataset partitioning stage and was not included in the reported total of 11,200 samples.

The source photographs were acquired using a DJI Phantom 4 Pro UAV equipped with an FC6310 RGB camera featuring a 1-inch CMOS sensor and a nominal resolution of 20 megapixels. The original photographs measured 5472 × 3648 pixels. Flights were conducted at an average altitude of approximately 50 m above ground level. These acquisition dimensions describe the original photographs and are distinct from the 224 × 224-pixel inputs used by the custom CNN.

The UAV images were acquired at Bakhmal district, Jizzakh (39.611814, 68.290907) during May–August, 2025, between 10:00 and 15:00. A total of 11,200 original RGB photographs with a resolution of 5472 × 3648 pixels were collected. From these photographs, 11,200 original, non-augmented image samples were prepared for the classification dataset. Differences in solar illumination angle, tree shadows, and terrain produced variation in image brightness. Images acquired in rain, fog, or insufficient illumination were excluded from the dataset. The source imagery is therefore relatively homogeneous in terms of the stated camera, RGB modality, and original pixel dimensions, while remaining heterogeneous in illumination, vegetation appearance, terrain, and background composition. A common pixel size does not establish uniform ground sampling distance, particularly over uneven terrain.

The images were supplied for research use under a written undertaking prohibiting disclosure and redistribution. The institute's associated research project is ongoing, and the dataset is consequently not publicly available at present. The authors are not authorized to release the imagery independently of the institute.

The samples were organized into four classes: healthy vegetation, stressed vegetation, damaged vegetation, and other areas. The damaged class includes vegetation with artificial or externally caused damage. The other areas class includes soil, rocks, roads, shadows, dry surfaces, and non-vegetation regions. The following expressions are not introduced as new mathematical formulations; they use standard supervised-learning notation to represent the image–label dataset, RGB image tensors, and class labels (Goodfellow et al., 2016). Table 2 summarizes the dataset and acquisition characteristics. The complete dataset is denoted as:


$$\begin{array}{l} D={\left\{\left(X_{i},y_{i}\right)\right\}}_{i=1}^{N} \end{array}$$

where *D* is the UAV image dataset, *X*_*i*_ is the *i*-th UAV image, *y*_*i*_ is its corresponding class label, and *N* is the total number of images. In this study, *N* = 11, 200.

**Table 2:** General description of the UAV image dataset.

Property	Description
Data source	Restricted institutional database of the Institute of Rainfed Agriculture of Republic of Uzbekistan: UAV RGB imagery
Number of study samples	11,200
Study environments	Forest farms and sloping mountain areas
Number of classes	4
Classes	Healthy vegetation, stressed vegetation, damaged vegetation, other areas
Main task	Green zone condition classification
Main challenges	Shadows, terrain variation, mixed vegetation, soil exposure, rocks, illumination changes, and visual similarity between stressed and damaged vegetation
UAV platform	DJI Phantom 4 Pro
Camera	FC6310; 20 MP RGB; 1-inch CMOS sensor
Original image dimensions	5472 × 3648 pixels
Average flight altitude	Approximately 50 m above ground level
Acquisition time of day	Predominantly 10:00–15:00
Lighting and weather	Natural daylight; clear or partly cloudy; variable shadows and illumination
Excluded conditions	Rain, fog, and insufficient illumination
Access conditions	Research use only under a written non-disclosure undertaking; public release currently prohibited

Since RGB images are used, each image is represented as:


$$\begin{array}{l} X_{i}\in R^{H\times W\times 3} \end{array}$$

where *H* is the image height, *W* is the image width, and 3 represents the red, green, and blue channels. The class label of each image is defined as:


$$\begin{array}{l} y_{i}\in \left\{0,1,2,3\right\} \end{array}$$

where, 0 → healthy vegetation, 1 → stressed vegetation, 2 → damaged vegetation, 3 → other areas.

The inclusion of the other areas class is important because UAV images collected from forest and mountain regions often contain visually confusing non-vegetation objects (Table 2). If such regions are not included as a separate class, the model may incorrectly classify soil, rocks, or shadows as damaged vegetation. Therefore, the four-class dataset structure allows the model to distinguish between real vegetation conditions and non-target background areas.

### 3.2 Image annotation

Each UAV image was assigned to one of the four predefined classes according to its dominant visual content. The annotation process was based on several visual indicators, including vegetation color, canopy density, texture structure, visible drying, damage-related canopy changes, and the presence of non-target objects, consistent with visual characteristics reported in UAV-based forest-health, standing-dead-tree, and tree-disease studies (Ecke et al., 2022; Jiang et al., 2023; Makhmudov et al., 2026). The annotation process can be expressed as:


$$\begin{array}{l} a\left(X_{i}\right)=y_{i} \end{array}$$

where *a*(·) denotes the annotation function, *X*_*i*_ is the input UAV image, and *y*_*i*_ is the assigned class label. Thus, the labeled dataset is represented as Goodfellow et al. (2016):


$$\begin{array}{l} D={\left\{\left(X_{i},a\left(X_{i}\right)\right)\right\}}_{i=1}^{11,200} \end{array}$$

Images with dense green cover, uniform vegetation texture, and no visible degradation symptoms were labeled as healthy vegetation. Images with reduced greenness, slight yellowing, weak canopy density, or early signs of stress were labeled as stressed vegetation. Images containing visible artificial damage, dry patches, broken vegetation structure, or severe local degradation were labeled as damaged vegetation. Images dominated by soil, rocks, shadows, roads, or other background objects were labeled as other areas.

To reduce subjectivity during annotation, representative samples from each class were selected and used as visual references. This step is important because the boundary between healthy and stressed vegetation, or between stressed and damaged vegetation, may not always be clearly visible in UAV images.

### 3.3 Image preprocessing

Before training, all images were preprocessed to obtain a consistent input format for CNN architectures. From each original 5472 × 3648 UAV photograph, one image patch representing its dominant target region was extracted, yielding the 11,200 samples used in this study. The resulting samples were then resized to a uniform size of 224 × 224 × 3 pixels before being supplied to the CNN. Thus, all 11,200 samples used in the experiments had the same input dimensions. Since CNN models require fixed-size input images, each UAV image was resized to a predefined spatial resolution. The input size may be 224 × 224, 256 × 256, or another model-specific resolution. The resizing operation is defined as:


$$\begin{array}{l} {\tilde{X}}_{i}=R\left(X_{i},H_{0},W_{0}\right) \end{array}$$

where *R*(·) is the resizing function, *X*_*i*_ is the original UAV image, *H*_0_ and *W*_0_ are the target height and width, and ${\tilde{X}}_{i}$ is the resized image.

After resizing, pixel normalization was applied (Zohirov et al., 2026). Since RGB pixel values are usually represented in the range [0, 255], normalization is defined as:


$$\begin{array}{l} X_{i}^{norm}=\frac{{\tilde{X}}_{i}}{255} \end{array}$$

After normalization, each pixel value satisfies:


$$\begin{array}{l} X_{i}^{norm}\left(u,v,c\right)\in \left[0,1\right] \end{array}$$

where *u* and *v* are pixel coordinates, and *c* denotes the RGB channel.

Preprocessing was performed carefully because vegetation stress and damage symptoms are often expressed through color and texture differences. Excessive color correction was avoided, since it may remove useful information such as yellowing, browning, reduced greenness, dry regions, and damaged texture patterns.

Spatial resizing and pixel-value normalization serve different purposes. Resizing establishes the spatial dimensions expected by the network, whereas division by 255 rescales RGB intensity values to the interval [0, 1] without changing the spatial dimensions. The source photograph dimensions should therefore be distinguished from the model input dimensions when interpreting the preprocessing pipeline.

The preprocessing pipeline was implemented in Python 3.13.11 using OpenCV 4.13.0.90. Image patches were resized to 224 × 224 pixels using cv2.resize() with INTER_AREA interpolation. Pixel intensities were then normalized to the [0,1] range by dividing RGB values by 255.

At this altitude, the nominal ground sampling distance of the FC6310 camera (13.2 mm sensor width, 8.8 mm focal length) is approximately 1.4 cm per pixel; the actual value varies with local terrain height. Figure 3 illustrates the original UAV image and its resized and normalized versions.

**Figure 3:**
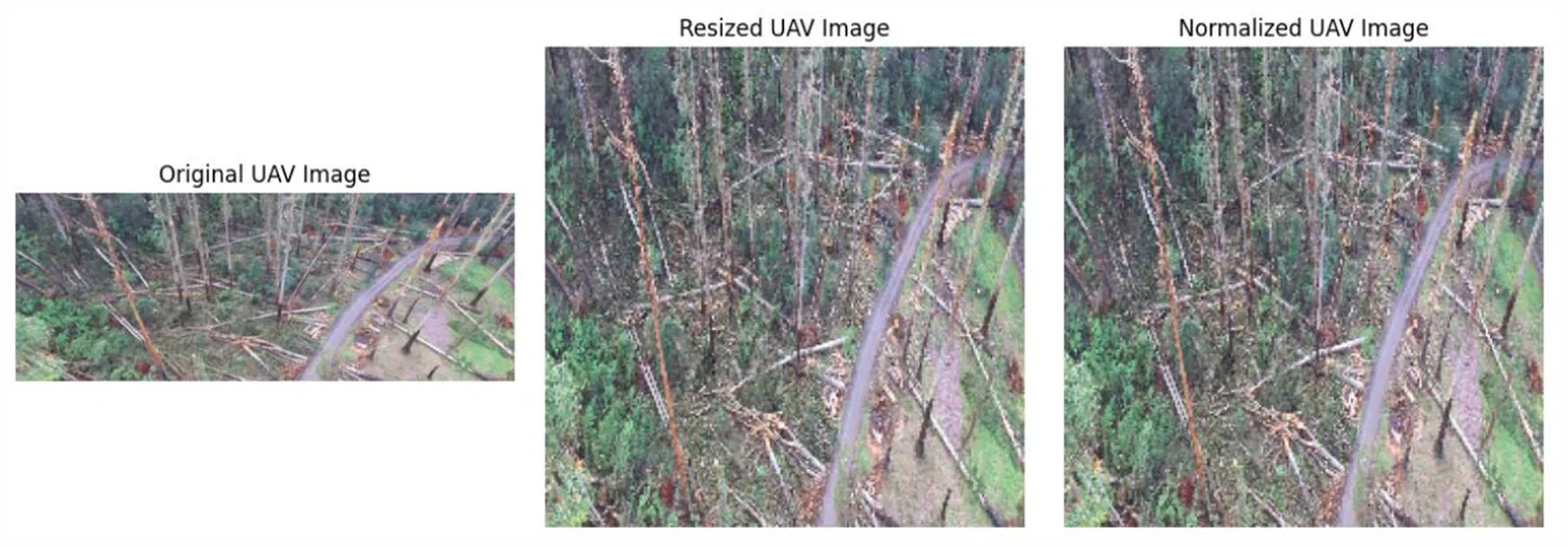
Original and preprocessed images of damaged forest from UAV.

### 3.4 Data augmentation

Data augmentation was applied only to the training subset. Augmented images were generated dynamically during training and were not counted as part of the original 11,200-image dataset. No augmentation was applied to the validation or test subsets. UAV images may vary due to camera angle, flight direction, sunlight position, shadows, wind, and terrain structure. Therefore, augmentation helps the model become more robust to real image acquisition conditions.

The augmented image is defined as:


$$\begin{array}{l} X_{i}^{aug}=T_{k}\left(X_{i}^{norm}\right) \end{array}$$

where *T*_*k*_(·) is the *k*-th augmentation transformation.

The augmentation transformation set is defined as:


$$T=\{T_{flip},T_{rot},T_{zoom},T_{shift},T_{bright},T_{contrast}\}$$

where *T*_*flip*_ denotes horizontal or vertical flipping, *T*_*rot*_ denotes small-angle rotation, *T*_*zoom*_ denotes random zooming, *T*_*shift*_ denotes spatial translation, *T*_*bright*_ denotes brightness adjustment, and *T*_*contrast*_ denotes contrast adjustment. The augmented training dataset is represented as:


$$D_{train}^{aug}=\{(T_{k}(X_{i}),y_{i})|(X_{i},y_{i})\in D_{train},T_{k}\in T\}$$

Augmentation was not applied to the validation and test sets. This ensures that model performance is evaluated on original unseen UAV images rather than artificially transformed samples. Figure 4 presents an original UAV image and examples of its augmented versions.

**Figure 4:**
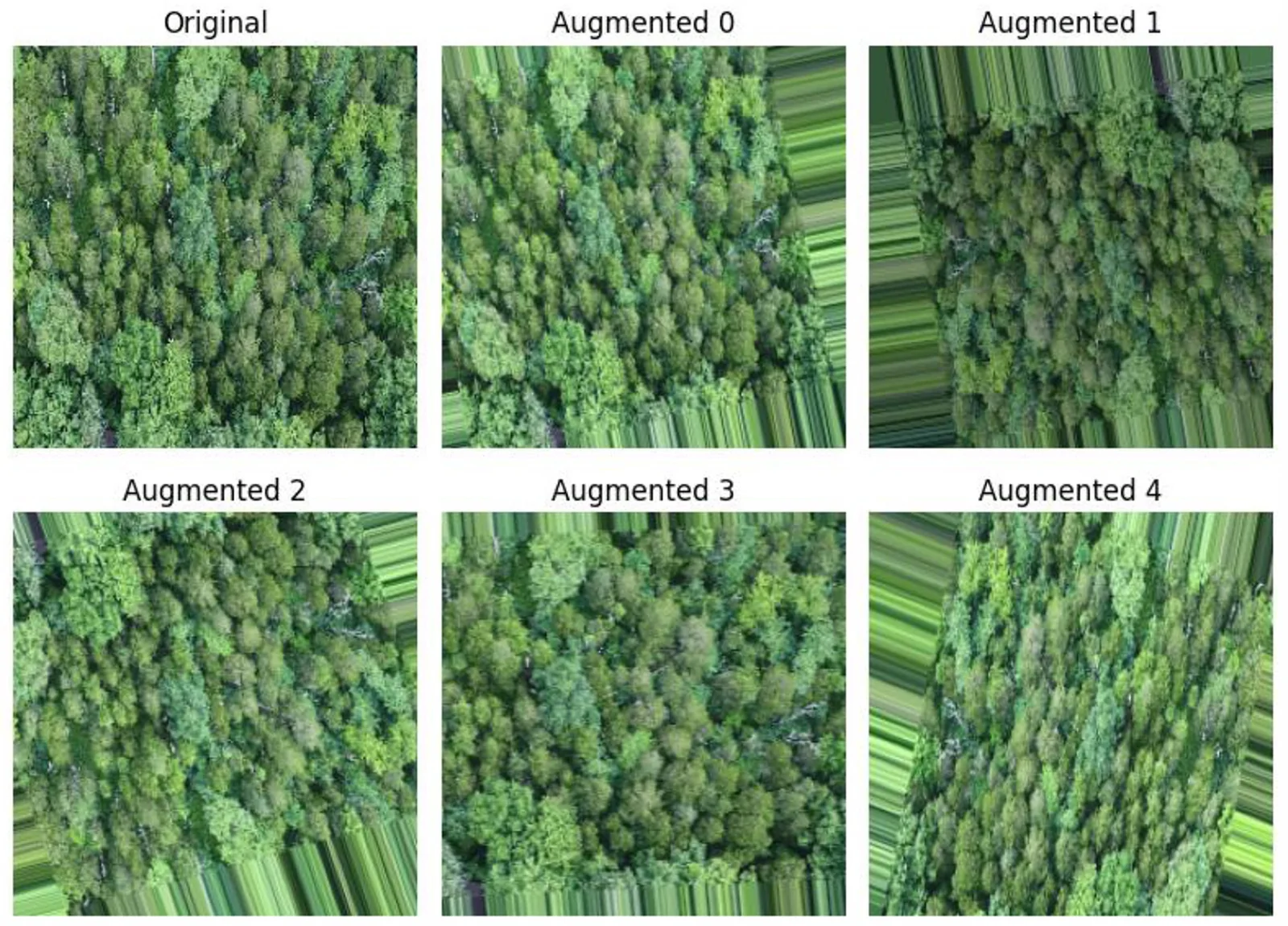
Results after augmentation.

### 3.5 Dataset partitioning

The complete dataset was divided into training, validation, and testing subsets. The training set was used to train CNN models and external classifiers. The validation set was used for early stopping and model selection. The test set was used only for final performance evaluation.

The dataset split is defined as:


$$\begin{array}{l} D=D_{train}\cup D_{val}\cup D_{test} \end{array}$$

The subsets are mutually exclusive:


$$\begin{array}{l} \;D_{train}\cap D_{val}=\phi \\ D_{train}\cap D_{test}=\phi \\ \;D_{val}\cap D_{test}=\phi \end{array}$$

A 70:15:15 split strategy was used. Since the dataset contains 11,200 images, the number of images in each subset is calculated as:


$$\begin{array}{l} N_{train}=0.70\times 11,200=7,840\\ \;N_{val}=0.15\times 11,200=1,680\\ \;N_{test}=0.15\times 11,200=1,680 \end{array}$$

For each class *k*, the nominal sample counts for a 70:15:15 allocation are expressed as:


$$\begin{array}{l} \;N_{train}^{\left(k\right)}=\left\lfloor 0.70N^{\left(k\right)}\right\rfloor \\ \;N_{val}^{\left(k\right)}=\left\lfloor 0.15N^{\left(k\right)}\right\rfloor \\ N_{test}^{\left(k\right)}=N^{\left(k\right)}-N_{train}^{\left(k\right)}-N_{val}^{\left(k\right)} \end{array}$$

where *N*^(*k*)^ is the total number of images belonging to class *k*, and *k*∈{0, 1, 2, 3} (metadata is illustrated in Table 3) .

**Table 3:** Dataset split used in the experiments.

Class	Training	Validation	Test	Total
Healthy vegetation	1,960	420	420	2,800
Stressed vegetation	1,960	420	420	2,800
Damaged vegetation	1,960	420	420	2,800
Other areas	1,960	420	420	2,800
Total	7,840	1,680	1,680	11,200

The dataset was balanced across the four classes, with 2,800 samples in each class. A stratified 70:15:15 split was applied to preserve the same class distribution in the training, validation, and test subsets. Accordingly, each class contained 1,960 training samples, 420 validation samples, and 420 test samples. Since the dataset was class-balanced, no additional class-balancing technique was required.

### 3.6 CNN-based feature extraction

A convolutional neural network is used as the main feature extraction mechanism. In the proposed framework, CNN models are not only considered as end-to-end classifiers, but also as feature extractors whose fully connected layers can be replaced by external machine learning classifiers.

The convolution operation in the *l*-th layer is defined as:


$$\begin{array}{l} Z_{i,j,k}^{\left(l\right)}=b_{k}^{\left(l\right)}+\overset{K_{h}-1}{\underset{u=0}{\sum }}\overset{K_{w}-1}{\underset{v=0}{\sum }}\overset{C_{l-1}}{\underset{c=1}{\sum }}W_{u,v,c,k}^{\left(l\right)A_{i+u,j+v,c}^{\left(l-1\right)}} \end{array}$$

where $Z_{i,j,k}^{\left(l\right)}$ is the convolution output at position *(i, j)* for the *k*-th feature map, $W_{u,v,c,k}^{\left(l\right)}$ is the convolution kernel weight, $b_{k}^{\left(l\right)}$ is the bias term, *A*^(*l*−1)^ is the output of the previous layer, *K*_*h*_ and *K*_*w*_ are the kernel height and width, and *C*_*l*−1_ is the number of input channels.

After convolution, the ReLU activation function is applied:


$$\begin{array}{l} A_{i,j,k}^{\left(l\right)}=\max\left(0,Z_{i,j,k}^{\left(l\right)}\right) \end{array}$$

Pooling is used to reduce the spatial size of feature maps and preserve important information. Max pooling is defined as:


$$\begin{array}{l} P_{i,j,k}^{\left(l\right)}=\underset{\left(u,v\right)\in \Omega }{\max}A_{si+u,sj+v,k}^{\left(l\right)} \end{array}$$

where Ω is the pooling window and *s* is the stride.

Average pooling is defined as:


$$\begin{array}{l} P_{i,j,k}^{\left(l\right)}=\frac{1}{|\Omega |}\underset{\left(u,v\right)\in \Omega }{\sum }A_{si+u,sj+v,k}^{\left(l\right)} \end{array}$$

In this study, different kernel sizes and pooling strategies are compared. The kernel set is defined as:


$$\begin{array}{l} K=\left\{3\times 3,5\times 5,7\times 7\right\} \end{array}$$

### 3.7 Feature vector construction

After convolution and pooling, the final feature maps must be converted into a one-dimensional feature vector. This step is important because external classifiers such as XGBoost, Random Forest, SVM, Logistic Regression require vector-based input.

Let the final convolutional feature map be:


$$\begin{array}{l} A^{\left(L\right)}\in R^{H_{f}\times W_{f}\times C_{f}} \end{array}$$

where *H*_*f*_, *W*_*f*_, and *C*_*f*_ denote the height, width, and number of channels of the final feature map.

Using the flatten operation, the feature map is converted into a vector:


$$\begin{array}{l} F_{i}^{flat}=Flatten\left(A_{i}^{\left(L\right)}\right) \end{array}$$

The dimensionality of the flattened feature vector is:


$$\begin{array}{l} d_{flat}=H_{f}\times W_{f}\times C_{f} \end{array}$$

Using global average pooling, each channel is averaged over its spatial dimensions:


$$\begin{array}{l} f_{i,k}^{gap}=\frac{1}{H_{f}W_{f}}\overset{H_{f}}{\underset{p=1}{\sum }}\overset{W_{f}}{\underset{q=1}{\sum }}A_{i,p,q,k}^{\left(L\right)} \end{array}$$

The global average pooling feature vector is expressed as:


$$\begin{array}{l} F_{i}^{gap}=\left[f_{i,1}^{gap},f_{i,2}^{gap},...,f_{i,C_{f}}^{gap}\right] \end{array}$$

The general CNN feature extraction process is written as:


$$\begin{array}{l} F_{i}=\phi_{\theta }\left(X_{i}^{prep}\right) \end{array}$$

where ϕ_θ_(·) denotes the CNN feature extractor with parameters θ, and *F*_*i*_ is the extracted feature vector.

### 3.8 Fully connected layer replacement strategy

In a conventional CNN model, the extracted features are passed to fully connected layers and then to a Softmax classifier. The predicted class is determined as:


$$\begin{array}{l} {ŷ}_{i}=\arg\underset{k}{\max}p_{i,k},k\in \left\{0,1,2,3\right\} \end{array}$$

where *p*_*i, k*_ is the predicted probability that image *X*_*i*_ belongs to class *k*.

In the proposed hybrid framework, the fully connected classification head is removed. The CNN is used as a feature extractor, and the extracted feature vector is passed to an external classifier:


$$\begin{array}{l} {ŷ}_{i}=g_{\psi }\left(F_{i}\right) \end{array}$$

where *g*_ψ_(·) denotes the external classifier and ψrepresents its parameters.

The classifier set is defined as:


$$\begin{array}{l} C=\left\{\text{XGBoost},\;\text{Random}\;\text{Forest},\;\text{SVM},\;\text{Logistic}\;\text{Regression}\right\} \end{array}$$

Thus, the general hybrid classification pipeline is:


$$\begin{array}{l} X_{i}\to \text{CNN}\;\text{feature}\;\text{extractor}\to F_{i}\to \text{external}\;\text{classifier}\to {ŷ}_{i} \end{array}$$

This strategy makes it possible to compare conventional CNN–Softmax models with hybrid CNN–machine learning models under the same dataset and evaluation conditions.

### 3.9 XGBoost classifier

XGBoost is used as the main external classifier in the proposed framework. It is based on gradient-boosted decision trees and builds an ensemble of weak learners to form a strong classification model.

Given the CNN-extracted feature vector *F*_*i*_, the XGBoost prediction is defined as:


$$\begin{array}{l} {ŷ}_{i}=\overset{T}{\underset{t=1}{\sum }}f_{t}\left(F_{i}\right),f_{t}\in F \end{array}$$

where *T* is the number of decision trees, *f*_*t*_ is the *t*-th tree, and F is the set of tree-based learners. The objective function is defined as Raximov et al. (2022):


$$\begin{array}{l} Obj=\overset{N}{\underset{i=1}{\sum }}l\left(y_{i},{ŷ}_{i}\right)+\overset{T}{\underset{t=1}{\sum }}\Omega \left(f_{t}\right) \end{array}$$

where *l*(*y*_*i*_, ŷ_*i*_) is the loss function and Ω(*f*_*t*_) is the regularization term. The regularization term is expressed as:


$$\begin{array}{l} \Omega \left(f\right)=\gamma T_{f}+\frac{1}{2}\lambda \overset{T_{f}}{\underset{j=1}{\sum }}w_{j}^{2} \end{array}$$

where *T*_*f*_ is the number of leaf nodes, *w*_*j*_ is the score of the *j*-th leaf, γ controls tree complexity, and λ is the regularization coefficient. At iteration *t*, the prediction is updated as:


$$\begin{array}{l} {ŷ}_{i}^{\left(t\right)}={ŷ}_{i}^{\left(t-1\right)}+f_{t}\left(F_{i}\right) \end{array}$$

Using the second-order Taylor approximation, the objective function at iteration *t* is written as:


$$\begin{array}{l} Obj^{\left(t\right)}\approx \overset{N}{\underset{i=1}{\sum }}\left[g_{i}f_{t}\left(F_{i}\right)+\frac{1}{2}h_{i}f_{t}^{2}\left(F_{i}\right)\right]+\Omega \left(f_{t}\right) \end{array}$$

where *g*_*i*_ and *h*_*i*_ are the first-order and second-order gradients:


$$\begin{array}{l} g_{i}=\frac{\partial l\left(y_{i},{ŷ}_{i}^{\left(t-1\right)}\right)}{\partial {ŷ}_{i}^{\left(t-1\right)}}\\ h_{i}=\frac{\partial^{2}l\left(y_{i},{ŷ}_{i}^{\left(t-1\right)}\right)}{\partial {\left({ŷ}_{i}^{\left(t-1\right)}\right)}^{2}} \end{array}$$

This mathematical structure allows XGBoost to use both gradient and curvature information during optimization.

### 3.10 Other external classifiers

In addition to XGBoost, several external classifiers are evaluated as alternative replacements for the fully connected classification head.

Random Forest constructs multiple decision trees and combines their predictions through majority voting:


$$\begin{array}{l} {ŷ}_{i}=mode\left\{h_{1}\left(F_{i}\right),h_{2}\left(F_{i}\right),...,h_{B}\left(F_{i}\right)\right\} \end{array}$$

where *B* is the number of trees and *h*_*b*_(·) is the prediction of the *b*-th tree.

Support Vector Machine finds an optimal separating hyperplane in the feature space. Its decision function is written as:


$$\begin{array}{l} f\left(F_{i}\right)=w^{T}F_{i}+b \end{array}$$

For multi-class classification, one-vs.-one or one-vs.-rest strategies are used.

Logistic Regression is used as a linear baseline classifier. For multi-class classification, the class probability is calculated by the Softmax function:


$$\begin{array}{l} p_{i,k}=\frac{\exp\left(w_{k}^{T}F_{i}+b_{k}\right)}{\sum_{r=0}^{3}\exp\left(w_{r}^{T}F_{i}+b_{r}\right)} \end{array}$$

The *k*-Nearest Neighbors classifier assigns the class label according to the most frequent class among the nearest feature vectors:


$$\begin{array}{l} {ŷ}_{i}=mode\left\{y_{j}|F_{j}\in N_{k}\left(F_{i}\right)\right\} \end{array}$$

where $N_{k}\left(F_{i}\right)$ is the set of *k* nearest neighbors of *F*_*i*_. These classifiers are not used as independent image feature extractors. Instead, they receive CNN-generated feature vectors and act as alternative classification heads (Table 4).

**Table 4:** The workflow details of this study.

Stage	Operation	Kernel	Output size
Input	RGB UAV image patch	—	224 × 224 × 3
Initial convolution	Conv + BN + ReLU	3 × 3, 32	224 × 224 × 32
Down-sampling	Max Pooling	2 × 2	112 × 112 × 32
Conv block 1	Conv + BN + ReLU	3 × 3, 64	112 × 112 × 64
Conv block 1	Max Pooling	2 × 2	56 × 56 × 64
Conv block 2	Conv + BN + ReLU	3 × 3, 128	56 × 56 × 128
Conv block 2	Max Pooling	2 × 2	28 × 28 × 128
Conv block 3	Conv + BN + ReLU	3 × 3, 256	28 × 28 × 256
Feature aggregation	Global Average Pooling	—	256
Feature vector	Deep feature representation	—	1 × 256
Classifier	Softmax/XGBoost/RF/SVM/LR	—	4 classes

### 3.11 Experimental configuration

For each CNN architecture *M*_*j*_, kernel configuration *K*_*s*_, pooling strategy *P*_*r*_, and classifier *C*_*q*_, an experimental configuration is defined as:


$$\begin{array}{l} E_{j,s,r,q}=\left(M_{j},K_{s},P_{r},C_{q}\right) \end{array}$$

where *M*_*j*_ denotes the CNN architecture, *K*_*s*_ denotes the kernel size, *P*_*r*_ denotes the pooling strategy, and *C*_*q*_ denotes the classifier.

The predicted output for each experimental configuration is written as:


$$\begin{array}{l} {ŷ}_{i}^{\left(j,s,r,q\right)}=C_{q}\left(\phi_{j,s,r}\left(X_{i}\right)\right) \end{array}$$

where ϕ_*j, s, r*_(·) denotes the CNN feature extractor of the custom CNN under the selected kernel and pooling configuration.

Within the custom CNN framework, this formulation enables a systematic comparison of convolutional kernel sizes, pooling strategies, feature vector construction methods, and external classifiers.

All experiments were implemented in Python 3.13.11 using TensorFlow 2.20.0 and Keras 3.13.1 for CNN construction and training, OpenCV 4.13.0.90 for image preprocessing, scikit-learn 1.8.0 for Random Forest, Support Vector Machine, and Logistic Regression, and XGBoost 3.1.3 for gradient-boosted classification. The custom CNN was trained using the Adam optimizer with an initial learning rate of 1 × 10^−4^, a batch size of 32, and a maximum of 50 epochs. Early stopping with a patience of 10 epochs was applied based on the validation loss to reduce the risk of overfitting. The experiments were performed on a workstation equipped with an NVIDIA GeForce RTX 3080 Ti GPU and an 11th Gen Intel Core i9-11900K CPU operating at 3.50 GHz. The principal hyperparameters of the external classifiers were kept fixed across the comparative experiments to ensure consistent evaluation conditions. The main experimental stages are summarized in Table 5, and the detailed layer operations and output dimensions are listed in Table 6.

**Table 5:** All stages of current research approach.

Stages	Focus	Purpose
S1	CNN–Softmax baseline	Obtaining baseline performance
S2	Kernel size variation	Analyzing receptive field effects
S3	Pooling comparison	To evaluate spatial down-sampling
S4	Flatten vs. GAP	Comparing feature vector construction
S5	Classifier replacement	Comparing CNN–Softmax and hybrid models
S6	Feature visualization	To interpret CNN internal representations

**Table 6:** Detailed operations and sizes.

Stages	Operation	Output size
Input	RGB image patch	224 × 224 × 3
Conv-1	Conv + BN + ReLU, 32 filters	224 × 224 × 32
Pool-1	Max pooling	112 × 112 × 32
Conv block-2	Conv + BN + ReLU, 64 filters	112 × 112 × 64
Pool-2	Max pooling	56 × 56 × 64
Conv block-3	Conv + BN + ReLU, 128 filters	56 × 56 × 128
Pool-3	Max pooling	28 × 28 × 128
Conv block-4	Conv + BN + ReLU, 256 filters	28 × 28 × 256
GAP	Global average pooling	1 × 256
Feature vector	CNN feature representation	256-D
Classifier	XGBoost/RF/SVM/LR	4 classes

### 3.12 Evaluation metrics

The performance of each model is evaluated using accuracy, precision, recall, F1-score, macro-F1, and confusion matrix. Accuracy is defined as Kuvandikov et al. (2026):


$$\begin{array}{l} Accuracy=\frac{TP+TN}{TP+TN+FP+FN} \end{array}$$

Precision is defined as:


$$\begin{array}{l} Precision=\frac{TP}{TP+FP} \end{array}$$

Recall is defined as:


$$\begin{array}{l} Recall=\frac{TP}{TP+FN} \end{array}$$

F1-score is defined as:


$$\begin{array}{l} F1=2\times \frac{Precision\times Recall}{Precision+Recall} \end{array}$$

For the four-class classification task, macro-F1 is calculated as:


$$\begin{array}{l} MacroF1=\frac{1}{K}\overset{K-1}{\underset{k=0}{\sum }}F1_{k} \end{array}$$

where *K* = 4 is the number of classes.

Special attention is given to the recall and F1-score values of the stressed vegetation and damaged vegetation classes. This is because missing these classes may reduce the practical usefulness of UAV-based green zone monitoring. The confusion matrix is also analyzed to identify whether stressed vegetation is misclassified as healthy vegetation or whether other areas are incorrectly detected as damaged vegetation.

### 3.13 Visualization of intermediate CNN operations

In addition to numerical evaluation, intermediate CNN operations are visualized to better understand how UAV images are transformed inside the network. Representative input images are passed through convolutional and pooling layers, and the resulting feature maps are analyzed.


$$\begin{array}{l} A_{k}^{\left(l\right)}=\phi \left(W_{k}^{\left(l\right)}\;*\;A^{\left(l-1\right)}+b_{k}^{\left(l\right)}\right) \end{array}$$

where $W_{k}^{\left(l\right)}$ is the convolutional kernel, *A*^(*l*−1)^ is the input feature map, $b_{k}^{\left(l\right)}$ is the bias term, and ϕ(·) is the activation function.

Feature map visualization helps to explain how CNN filters respond to vegetation texture, color degradation, dry patches, canopy discontinuity, and non-target background objects (Onishi and Ise, 2021). Pooling outputs are also visualized to observe how spatial information is compressed and whether damage-related patterns are preserved after down-sampling. Figure 5 shows selected feature maps after convolution, batch normalization, and ReLU activation. Figure 6 illustrates a final convolutional feature map and its associated 256-dimensional GAP representation. Figures 7, 8 show selected feature maps from the first and second max-pooling layers, respectively. Figure 9 presents selected final convolutional feature maps for the other-areas sample. Figures 10, 11 illustrate the transformation of representative other-areas and damaged-vegetation samples, respectively, into final convolutional feature maps and GAP feature vectors.

**Figure 5:**
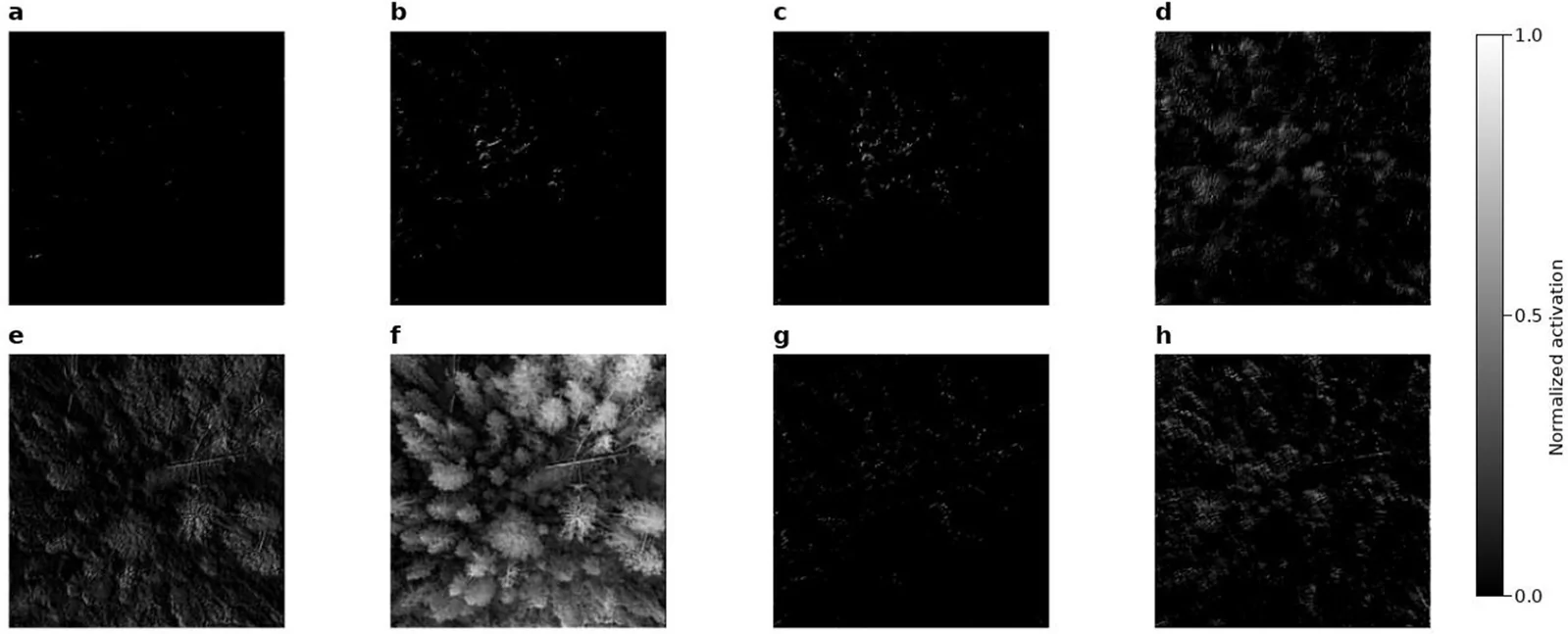
Selected feature maps after convolution, batch normalization, and ReLU activation. **(a–h)** Show feature maps 1–8, respectively. The colorbar indicates normalized activation values.

**Figure 6:**
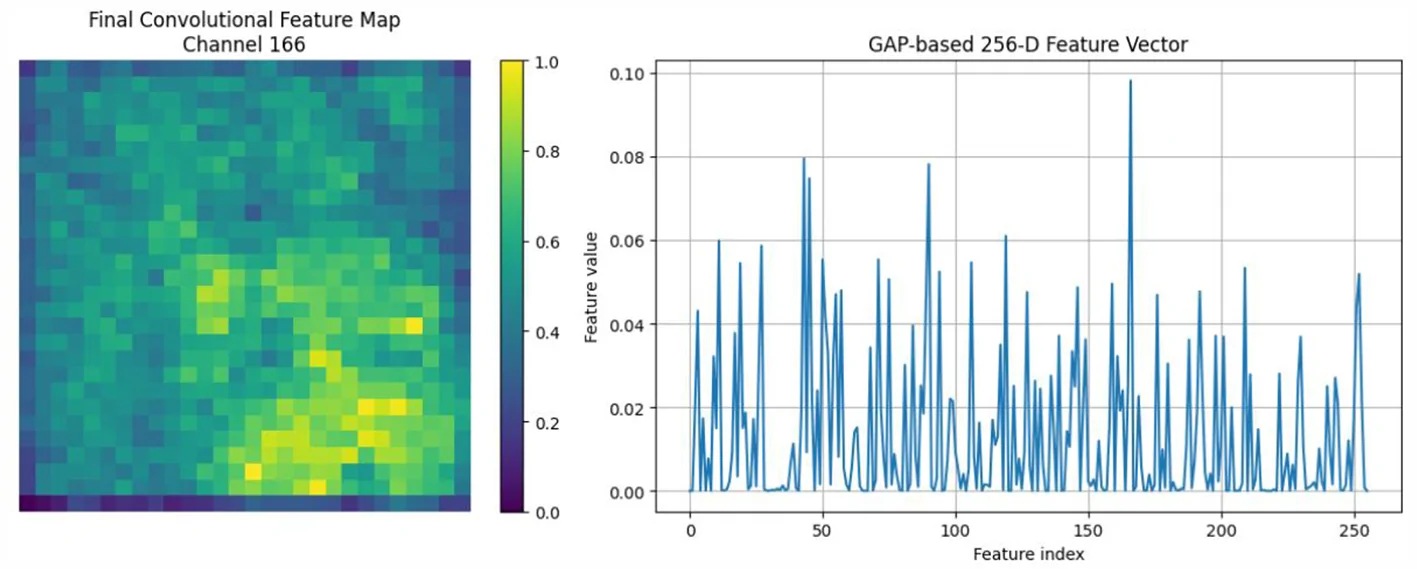
Final convolutional representation and global average pooling output. **(Left)** The spatial activation map of channel 166. **(Right)** The 256-dimensional GAP feature vector; the horizontal axis denotes the feature index and the vertical axis denotes the GAP activation value.

**Figure 7:**
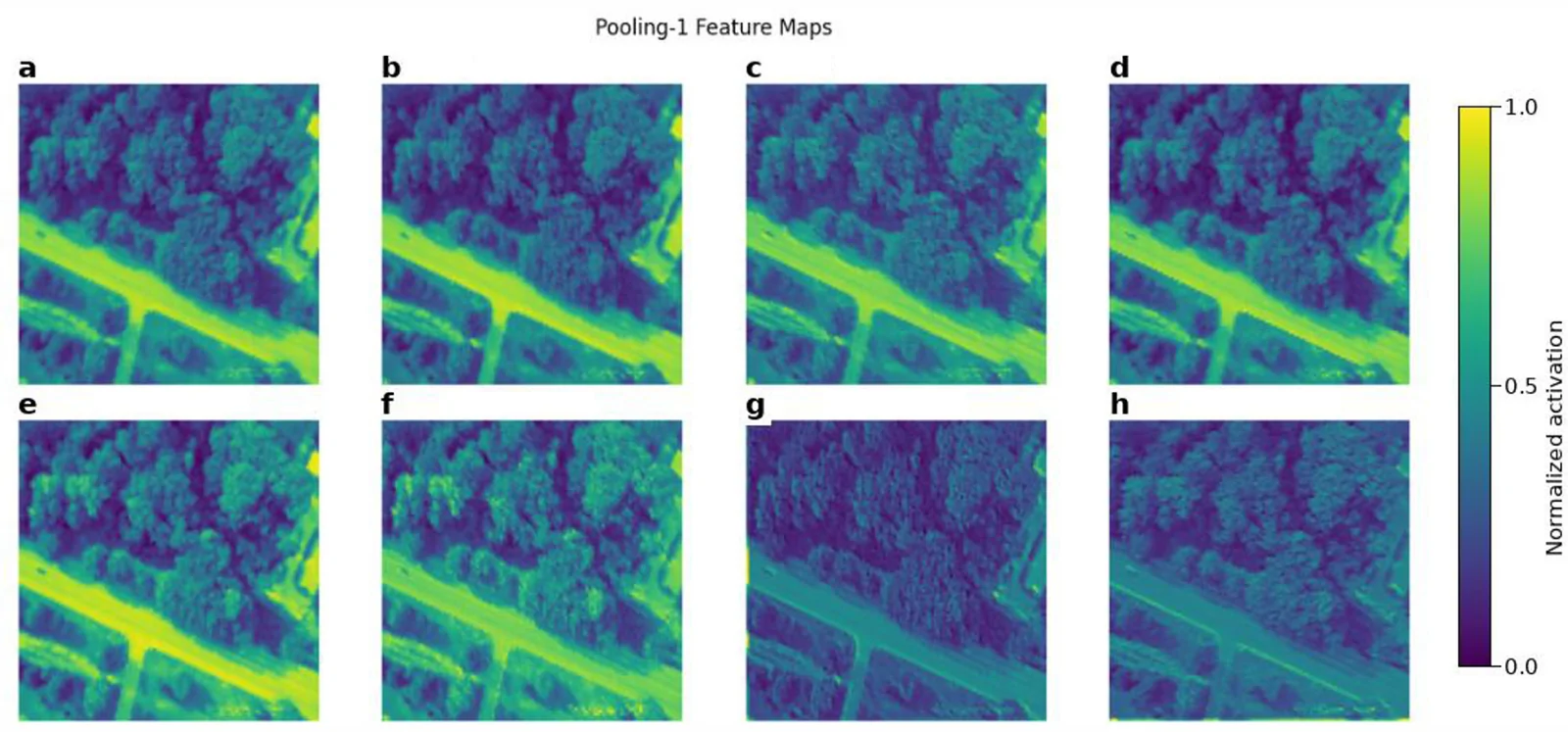
Feature maps from the first max-pooling layer for a sample labeled as other areas. **(a–h)** Correspond to channels 19, 1, 21, 29, 30, 5, 15, and 9, respectively. The colorbar indicates normalized activation values.

**Figure 8:**
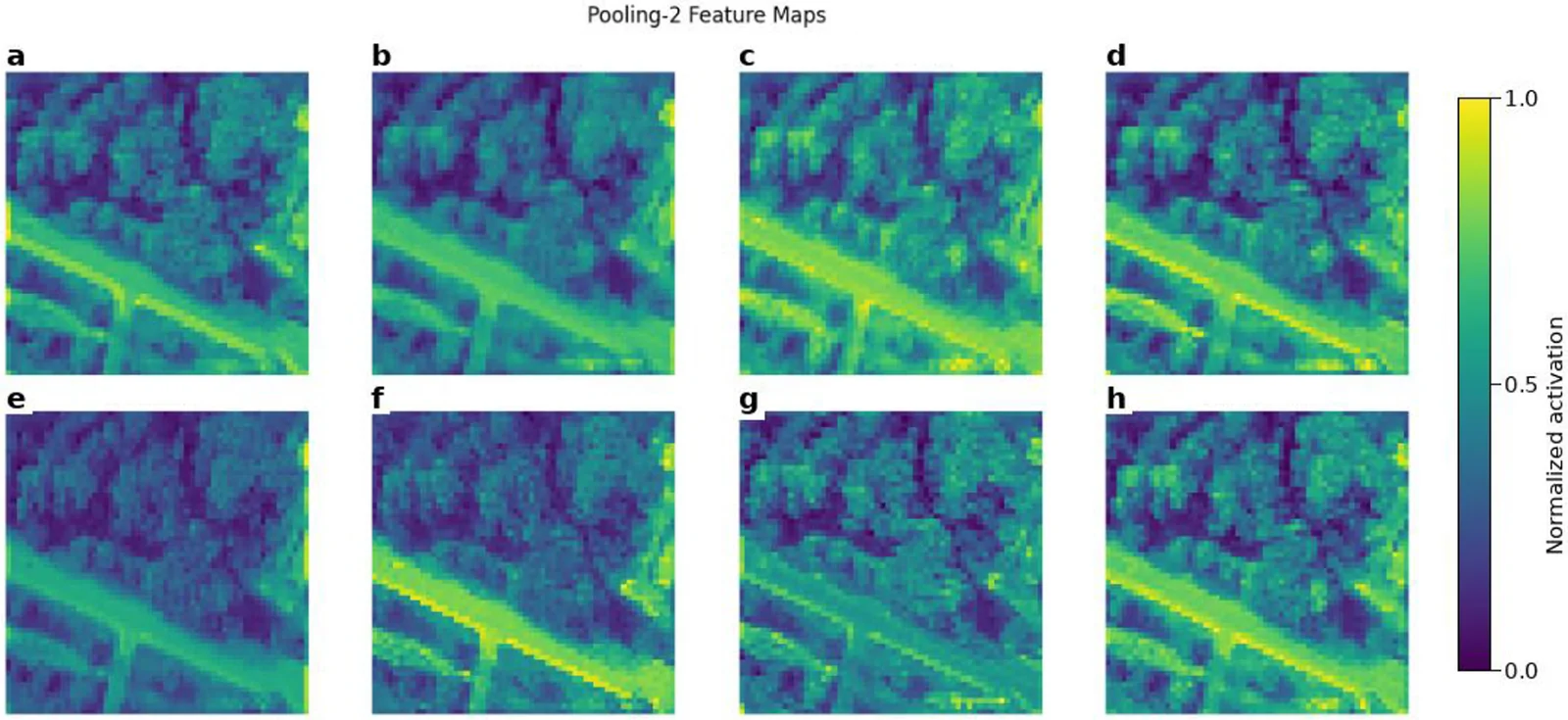
Feature maps from the second max-pooling layer for the same other-areas sample. **(a–h)** Correspond to channels 3, 29, 10, 15, 4, 18, 46, and 32, respectively. The colorbar indicates normalized activation values.

**Figure 9:**
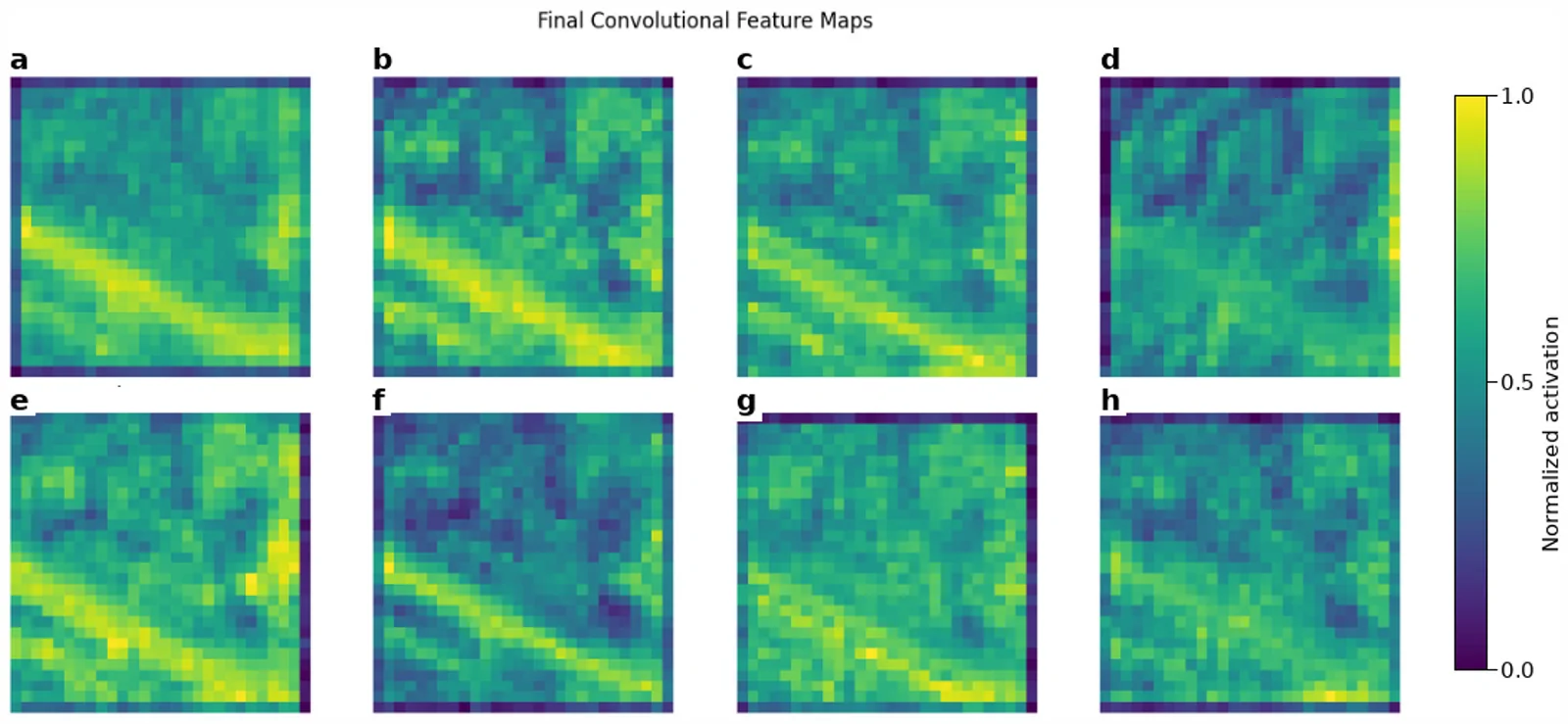
Selected final convolutional feature maps for the other-areas sample. **(a–h)** Correspond to channels 178, 142, 170, 39, 96, 225, 135, and 133, respectively. The colorbar indicates normalized activation values.

**Figure 10:**
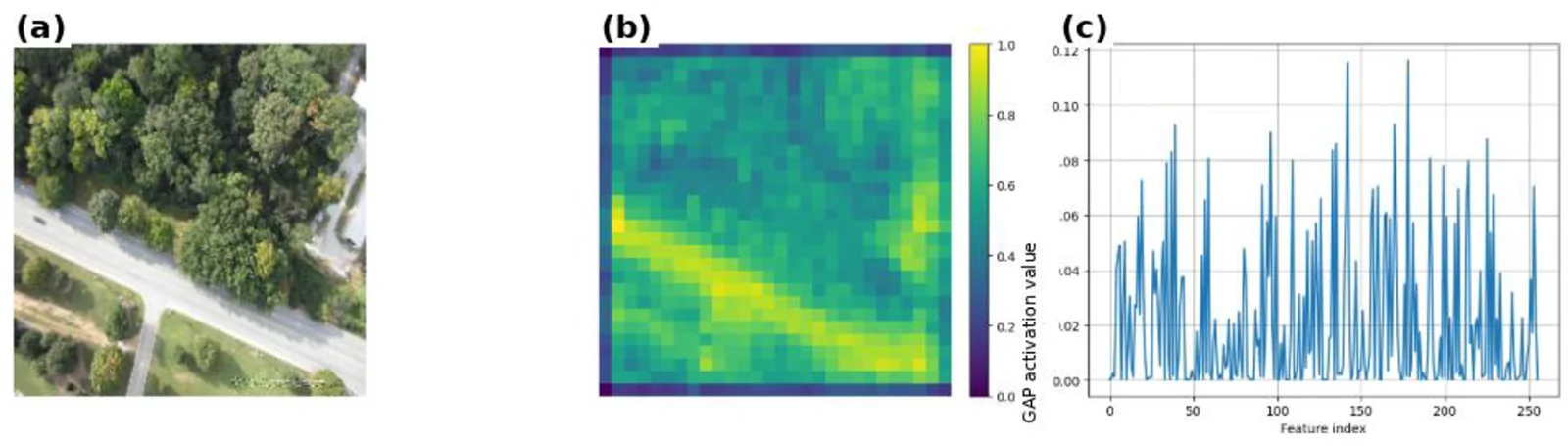
Transformation of an other-areas sample into its CNN representation: **(a)** original RGB UAV image patch, **(b)** final convolutional feature map for channel 178, and **(c)** 256-dimensional GAP feature vector. In **(c)**, the horizontal axis denotes the feature index and the vertical axis denotes the GAP activation value.

**Figure 11:**
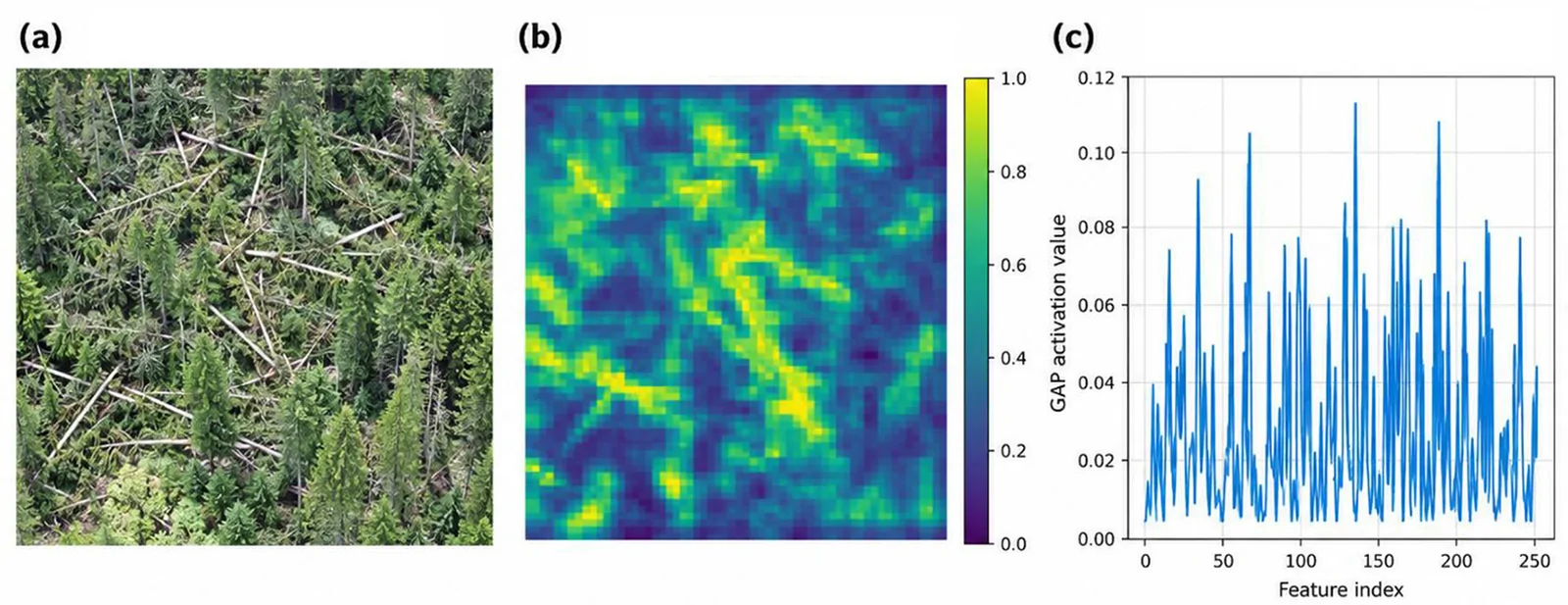
Transformation of a damaged-vegetation sample into its CNN representation. **(a)** Original RGB UAV image patch, **(b)** final convolutional feature map channel 178, **(c)** GAP based 256-D feature vector.

To further illustrate class-dependent CNN behavior, an additional visualization was prepared for a representative damaged-vegetation sample. This example complements the previous visualization of the “other areas” class and shows how the network represents damage-related patterns in the final convolutional and GAP-based feature spaces.

## 4 Experimental design and model configurations

In this section, we describe the experimental design used to evaluate the proposed CNN–external classifier framework. The experiments focus on how CNN architectural components and classifier replacement strategies affect UAV-based green zone condition classification. The main comparison is performed between conventional CNN–Softmax models and hybrid CNN–machine learning models for four classes: healthy vegetation, stressed vegetation, damaged vegetation, and other areas.

### 4.1 Experimental scenarios

We organized the experiments into six main scenarios. Firstly, conventional CNN–Softmax models are evaluated as baseline models. Secondly, different convolutional kernel sizes are tested to analyze the effect of receptive field size. Then, several pooling strategies are compared to evaluate how spatial down-sampling affects feature representation. Next stage, flatten and global average pooling are compared for feature vector construction. After that, fully connected layers are replaced with external classifiers. Finally, intermediate CNN outputs are visualized to interpret how UAV images are transformed inside the network.

### 4.2 Custom CNN architecture

The proposed custom CNN receives a 224 × 224 × 3 UAV image patch as input. The architecture consists of convolution, batch normalization, ReLU activation, max pooling, additional convolution blocks, global average pooling, feature vector generation, and an external classifier. The architecture is illustrated in Figure 12, and the layer dimensions are detailed in Table 6.

**Figure 12:**
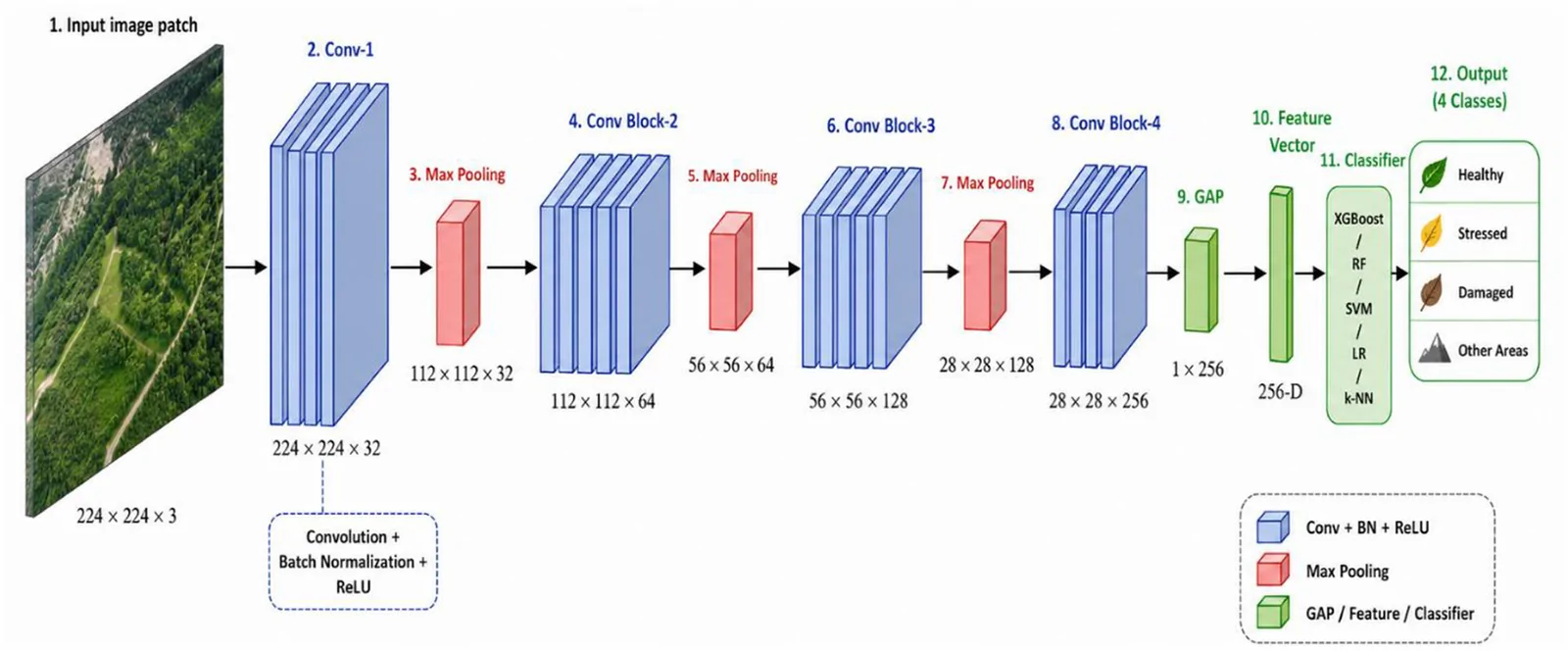
Proposed CNN architecture with an external classifier for four-class green zone condition classification.

This structure produces a compact 256-dimensional feature vector, which is then passed to external classifiers.

### 4.3 Kernel and pooling variations

We compare three convolutional kernel sizes: 3 × 3, 5 × 5, and 7 × 7. The 3 × 3 kernel captures fine texture and small damaged regions, the 5 × 5 kernel captures medium-scale vegetation changes, and the 7 × 7 kernel captures broader spatial patterns.

We also compare max pooling, average pooling, global average pooling, and global max pooling. Max pooling preserves the strongest local responses, while average pooling preserves smoother regional patterns. Global average pooling produces compact feature vectors and reduces the need for fully connected layers.

**Component Compared options**

Kernel size 3 × 3, 5 × 5, 7 × 7

Pooling type Max Pooling, Average Pooling, GAP, Global Max Pooling.

### 4.4 Fully connected layer replacement

In conventional CNN models, the final classification is performed by fully connected layers and a Softmax classifier. In the proposed framework, we remove this classification head and replace it with external machine learning classifiers.

The conventional structure is:

CNN feature extractor → Fully connected layers → Softmax → Class prediction

The proposed hybrid structure is:

CNN feature extractor → Feature vector → External classifier → Class prediction

The tested external classifiers include XGBoost, Random Forest, Support Vector Machine, and Logistic Regression.

## 5 Comparative results of fully connected layer replacement

After extracting 256-dimensional feature vectors from the global average pooling layer of the CNN model, we replaced the conventional fully connected Softmax classification head with four external classifiers: XGBoost, Random Forest, Support Vector Machine, and Logistic Regression. We trained all classifiers using the same CNN-extracted features and evaluated them on the same test set to ensure a fair comparison. The main purpose of this stage was to determine whether external classifiers can improve the classification of UAV-based green zone conditions compared with the conventional CNN–Softmax structure. The comparison focused on four classes: healthy vegetation, stressed vegetation, damaged vegetation, and other areas. Since stressed and damaged vegetation classes are more important for green zone monitoring, we considered macro-F1 and damaged-class performance as more informative than accuracy alone.

The results show that replacing the fully connected Softmax head with external classifiers improves the classification performance in most cases. The CNN–XGBoost model achieved the highest overall performance, with an accuracy of 0.936 and a macro-F1 score of 0.929. This indicates that XGBoost can better separate complex CNN-extracted vegetation features, especially when the visual differences between stressed and damaged vegetation are subtle. Random Forest also improved the baseline CNN–Softmax result and achieved a macro-F1 score of 0.912. This suggests that ensemble tree-based classifiers can effectively handle the non-linear feature structure extracted by the CNN. The CNN–SVM model produced competitive results, but its performance was slightly lower than XGBoost and Random Forest. Logistic Regression showed the lowest performance among the external classifiers, which indicates that the extracted CNN features are not fully linearly separable.

The class-wise F1-scores are reported in Table 7. The class-wise results show that all models classify healthy vegetation and other areas more accurately than stressed and damaged vegetation. This is expected because healthy vegetation and non-target areas usually have clearer visual characteristics, while stressed and damaged vegetation may share similar color and texture patterns. The CNN–XGBoost model achieved the best F1-score for stressed vegetation and damaged vegetation, with values of 0.908 and 0.925, respectively. This result is important because the main goal of the proposed framework is to improve the detection of early stress and visible damage in green zones. Compared with the conventional CNN–Softmax model, CNN–XGBoost improved the macro-F1 score from 0.893 to 0.929. The damaged vegetation F1-score also increased from 0.876 to 0.925. This improvement shows that the fully connected Softmax head is not always the most effective classification module for UAV-based vegetation damage detection. In contrast, XGBoost can use the CNN-generated feature vector more effectively by modeling non-linear decision boundaries between visually similar classes. The overall classification results are presented in Table 8.

**Table 7:** Class-wise F1-score comparison of CNN-based classification heads.

Model	Healthy vegetation	Stressed vegetation	Damaged vegetation	Other areas
CNN–Softmax	0.925	0.861	0.876	0.910
CNN–XGBoost	**0.954**	**0.908**	**0.925**	**0.930**
CNN–Random Forest	0.941	0.889	0.907	0.911
CNN–SVM	0.932	0.874	0.892	0.906
CNN–Logistic Regression	0.901	0.824	0.842	0.877

Bold values indicate the best performance for each metric.

**Table 8:** Comparative results of CNN feature extractor with different classification heads.

Model	Accuracy	Precision	Recall	F1-score	Macro-F1
CNN–Softmax	0.902	0.899	0.902	0.900	0.893
CNN–XGBoost	**0.936**	**0.934**	**0.936**	**0.935**	**0.929**
CNN–Random Forest	0.921	0.918	0.921	0.919	0.912
CNN–SVM	0.910	0.907	0.910	0.908	0.901
CNN–Logistic Regression	0.874	0.868	0.874	0.870	0.861

Bold values indicate the best performance for each metric.

## 6 Ablation study of CNN architectural components

To further analyze the proposed framework, we conducted an ablation study on the main CNN architectural components. The purpose of this experiment was to determine how convolutional kernel size, pooling strategy, and feature vector construction affect UAV-based green zone condition classification. Since the previous experiment showed that CNN–XGBoost achieved the best overall performance, we used XGBoost as the final classifier in this stage.

### 6.1 Effect of convolutional kernel size

We first evaluated the effect of different convolutional kernel sizes. Three kernel configurations were compared: 3 × 3, 5 × 5, and 7 × 7. The same CNN structure and XGBoost classifier were used in all cases.

The results show that the 3 × 3 kernel achieved the best performance among the tested configurations. This indicates that fine local features are important for identifying stressed and damaged vegetation in UAV images. Small kernels were more effective in capturing local texture changes, dry patches, small damaged regions, and subtle color variations. The 5 × 5 kernel produced slightly lower results, while the 7 × 7 kernel showed a clearer performance decrease. This suggests that larger kernels may capture broader spatial structures but may also reduce sensitivity to small vegetation damage patterns. Table 9 presents the effect of convolutional kernel size on classification performance.

**Table 9:** Effect of convolutional kernel size on classification performance.

Kernel size	Accuracy	Precision	Recall	F1-score	Macro-F1
3 × 3	**0.936**	**0.934**	**0.936**	**0.935**	**0.929**
5 × 5	0.927	0.924	0.927	0.925	0.918
7 × 7	0.908	0.904	0.908	0.905	0.897

Bold values indicate the best performance for each metric.

### 6.2 Effect of pooling strategy

We then compared different pooling strategies: max pooling, average pooling, global average pooling, and global max pooling. The purpose of this experiment was to analyze how spatial down-sampling affects the extracted feature representation.

The best result was obtained when max pooling was used in intermediate layers and global average pooling was used before the classifier. Max pooling helped preserve strong local activations associated with damaged or stressed regions, while global average pooling produced a compact and stable feature vector for XGBoost. Average pooling produced lower results because it smoothed local damage-related responses. Global max pooling emphasized only the strongest activation values and may have ignored broader vegetation context. Table 10 compares the classification performance obtained with the evaluated pooling strategies.

**Table 10:** Effect of pooling strategy on classification performance.

Pooling strategy	Accuracy	Precision	Recall	F1-score	Macro-F1
Max pooling + GAP	**0.936**	**0.934**	**0.936**	**0.935**	**0.929**
Average pooling + GAP	0.918	0.915	0.918	0.916	0.909
Global max pooling	0.911	0.908	0.911	0.909	0.902
Global average pooling	0.926	0.923	0.926	0.924	0.917

Bold values indicate the best performance for each metric.

### 6.3 Flatten vs. global average pooling

We also compared flatten and global average pooling for feature vector construction. Flatten produced a high-dimensional feature vector, while global average pooling generated a compact 256-dimensional representation.

The global average pooling representation outperformed the flatten representation. Although flatten preserved more spatial information, it produced a very high-dimensional feature vector, which increased the risk of overfitting and made external classifier training less stable. In contrast, global average pooling reduced the feature map into a compact 256-dimensional vector and provided a more generalizable representation for XGBoost. This result supports the use of global average pooling in the proposed hybrid architecture. Table 11 compares flatten and global average pooling in terms of feature dimensionality and classification performance.

**Table 11:** Comparison of flatten and global average pooling feature representations.

Feature construction method	Feature dimension	Accuracy	Precision	Recall	F1-score	Macro-F1
Flatten	200,704-D	0.913	0.909	0.913	0.910	0.903
Global average pooling	256-D	**0.936**	**0.934**	**0.936**	**0.935**	**0.929**

Bold values indicate the best performance for each metric.

### 6.4 Combined best configuration

Based on the ablation results, the best-performing configuration used a 3 × 3 convolutional kernel, max pooling in intermediate layers, global average pooling for feature aggregation, and XGBoost as the final classifier.

The results indicate that the best performance was achieved when the CNN extracted compact but discriminative visual features and XGBoost performed the final classification. This combination allowed the model to capture local vegetation damage patterns while maintaining a stable decision boundary between visually similar classes. The selected architectural components of the best-performing configuration are summarized in Table 12.

**Table 12:** Best configuration identified from the ablation study.

Component	Selected configuration
Input image size	224 × 224 × 3
Kernel size	3 × 3
Intermediate pooling	Max pooling
Feature aggregation	Global average pooling
Feature vector size	256-D
Final classifier	XGBoost
Output classes	Healthy, stressed, damaged, other areas

## 7 Discussion

The experimental results show that replacing the conventional fully connected Softmax head with external classifiers improves UAV-based green zone condition classification. The baseline CNN–Softmax model achieved an accuracy of 0.902 and a macro-F1 score of 0.893, while the proposed CNN–XGBoost model increased these values to 0.936 and 0.929, respectively. This means that the replacement of the fully connected head with XGBoost improved accuracy by 3.4 percentage points and macro-F1 by 3.6 percentage points. More importantly, the F1-score for the damaged vegetation class increased from 0.876 to 0.925, which confirms that XGBoost better separates visually complex damage-related features extracted by the CNN. Among the tested classifiers, XGBoost produced the best result, followed by Random Forest, SVM, and Logistic Regression. Random Forest achieved a macro-F1 score of 0.912, which shows that tree-based ensemble methods are suitable for CNN-extracted feature vectors. SVM obtained a macro-F1 score of 0.901, indicating moderate class separability in the CNN feature space. Logistic Regression produced the weakest result, with a macro-F1 score of 0.861, which suggests that the extracted features are not fully linearly separable. This difference is especially important for stressed and damaged vegetation classes, where the visual patterns are closer to each other. The ablation study also confirmed that CNN architectural choices strongly affect the final classification result. The 3 × 3 kernel achieved the best macro-F1 score of 0.929, while 5 × 5 and 7 × 7 kernels achieved 0.918 and 0.897, respectively. This result indicates that damaged vegetation in UAV images is better represented by fine local features such as texture irregularities, dry patches, color degradation, and small canopy breaks. Larger kernels captured broader spatial context, but they reduced sensitivity to small damage patterns.

The comparison between flatten and global average pooling was particularly important. Flatten produced a 200,704-dimensional feature vector, while global average pooling produced only a 256-dimensional vector. Despite this large reduction in dimensionality, global average pooling improved macro-F1 from 0.903 to 0.929. This shows that a compact feature vector can represent UAV vegetation patterns more effectively than a very high-dimensional flattened representation. It also reduces overfitting risk and makes external classifier training more stable.

Related UAV RGB studies provide context for these results. Onishi and Ise (2021) combined tree-crown segmentation with CNN classification for seven tree classes. Their fall-season ResNet152 model achieved an overall accuracy of 0.976 and an F1-score of 0.962. In comparison, the present CNN–XGBoost model achieved an accuracy of 0.936 and a macro-F1 score of 0.929 for four vegetation-condition classes. The tasks, datasets, class definitions, and evaluation protocols differ, so these values cannot establish a performance ranking. Their use of Guided Grad-CAM also highlights a distinction between class-specific explanations and the intermediate feature-map visualizations presented here.

For a more directly damage-related task, Jiang et al. (2023) detected standing dead trees in UAV RGB imagery using an improved Faster R-CNN with a Swin Transformer backbone and multi-scale feature processing. They reported an average precision of 95.9% at an IoU threshold of 0.5 and a recall of 92.1%. Those object-detection metrics are not directly comparable with the image-classification accuracy and macro-F1 reported here. Their work emphasizes localization and multi-scale representation, whereas the present study examines how kernel size, pooling, feature aggregation, and classification-head selection affect vegetation-condition classification. The observed 3.4-percentage-point accuracy gain is therefore supported by the internal CNN–Softmax comparison, rather than by a claim of superiority over these external studies.

The present findings are also consistent with the CNN–XGBoost studies discussed in Section 2. Ren et al. (2017) demonstrated that CNN-extracted features can be effectively classified using XGBoost on standard image-classification datasets, supporting the general two-stage feature-extraction and classification strategy. Jiao et al. (2021) further improved this framework through adaptive particle swarm optimization and reported classification accuracies of 99.60% on MNIST, 91.98% on CIFAR-10, and 66.35% on CIFAR-100. These results are not directly comparable with the present UAV vegetation-condition task because the datasets, image characteristics, class definitions, and evaluation protocols differ. Nevertheless, they provide methodological support for replacing the conventional CNN classification head with XGBoost. In the present study, this strategy is evaluated specifically for UAV-based vegetation-condition classification and is further analyzed in terms of kernel size, pooling strategy, feature aggregation, and alternative classification heads.

Several limitations constrain the interpretation of these findings. The evaluation uses a restricted institutional dataset, and transfer to independently acquired imagery has not been established by the results reported here. The acquisition protocol excludes rain, fog, and low-light conditions, limiting conclusions about performance in those settings. Moreover, the available feature-map visualizations describe intermediate responses but do not by themselves demonstrate which spatial regions caused a particular XGBoost prediction. These limitations should be considered when applying the model outside the evaluated data.

The evaluated task assigns one condition label to an input image or patch. It does not produce pixel-level damage masks or bounding boxes around individual damaged trees. Consequently, the classification results support recognition of vegetation condition in the evaluated samples, but do not establish the accuracy of damage-area estimation or object localization. Such spatial outputs would require separate annotations and evaluation.

## 8 Conclusions

This study evaluated a custom CNN-based framework for classifying healthy, stressed, damaged vegetation, and other areas in UAV RGB imagery by examining convolutional kernel size, pooling strategy, feature aggregation, and alternative classification heads. The best-performing configuration combined 3 × 3 convolutional kernels, intermediate max pooling, global average pooling, a 256-dimensional feature representation, and XGBoost as the final classifier. This configuration achieved an accuracy of 0.936 and a macro-F1 score of 0.929, while the damaged-vegetation class reached an F1-score of 0.925.

The results demonstrate the practical potential of combining compact CNN-derived representations with an external tree-based classifier for UAV-based vegetation-condition assessment. The proposed framework can support automated differentiation of healthy, stressed, damaged, and non-vegetation areas from high-resolution RGB UAV imagery.

Future work will focus on validation using independently acquired UAV datasets, a wider range of environmental and illumination conditions, and additional vegetation types. Further extensions should also investigate pixel-level damage segmentation, object localization, and more advanced explainability methods for identifying the image regions that contribute directly to the final classification decision.

## Data Availability

The data analyzed in this study are not publicly available because they were provided by the Research Institute of Rainfed Agriculture of the Republic of Uzbekistan under a written non-disclosure undertaking. Requests to access the data should be directed to the corresponding author and are subject to permission from the institute.
